# Influenza Human Monoclonal Antibody 1F1 Interacts with Three Major Antigenic Sites and Residues Mediating Human Receptor Specificity in H1N1 Viruses

**DOI:** 10.1371/journal.ppat.1003067

**Published:** 2012-12-06

**Authors:** Tshidi Tsibane, Damian C. Ekiert, Jens C. Krause, Osvaldo Martinez, James E. Crowe, Ian A. Wilson, Christopher F. Basler

**Affiliations:** 1 Department of Microbiology, Mount Sinai School of Medicine, New York City, New York, United States of America; 2 Department of Molecular Biology and the Skaggs Institute for Chemical Biology, The Scripps Research Institute, La Jolla, California, United States of America; 3 Department of Pediatrics, Vanderbilt University Medical Center, Nashville, Tennessee, United States of America; 4 Department of Microbiology and Immunology, Vanderbilt University Medical Center, Nashville, Tennessee, United States of America; Johns Hopkins University - Bloomberg School of Public Health, United States of America

## Abstract

Most monoclonal antibodies (mAbs) to the influenza A virus hemagglutinin (HA) head domain exhibit very limited breadth of inhibitory activity due to antigenic drift in field strains. However, mAb 1F1, isolated from a 1918 influenza pandemic survivor, inhibits select human H1 viruses (1918, 1943, 1947, and 1977 isolates). The crystal structure of 1F1 in complex with the 1918 HA shows that 1F1 contacts residues that are classically defined as belonging to three distinct antigenic sites, Sa, Sb and Ca_2_. The 1F1 heavy chain also reaches into the receptor binding site (RBS) and interacts with residues that contact sialoglycan receptors and determine HA receptor specificity. The 1F1 epitope is remarkably similar to the previously described murine HC63 H3 epitope, despite significant sequence differences between H1 and H3 HAs. Both antibodies potently inhibit receptor binding, but only HC63 can block the pH-induced conformational changes in HA that drive membrane fusion. Contacts within the RBS suggested that 1F1 may be sensitive to changes that alter HA receptor binding activity. Affinity assays confirmed that sequence changes that switch the HA to avian receptor specificity affect binding of 1F1 and a mAb possessing a closely related heavy chain, 1I20. To characterize 1F1 cross-reactivity, additional escape mutant selection and site-directed mutagenesis were performed. Residues 190 and 227 in the 1F1 epitope were found to be critical for 1F1 reactivity towards 1918, 1943 and 1977 HAs, as well as for 1I20 reactivity towards the 1918 HA. Therefore, 1F1 heavy-chain interactions with conserved RBS residues likely contribute to its ability to inhibit divergent HAs.

## Author Summary

Influenza infection kills thousands of people every year and causes major pandemics every few decades. The most lethal outbreak of influenza known was the 1918 H1N1 influenza pandemic that killed an estimated 20 to 100 million people. The 1918 virus was likely introduced into the human population from birds. We previously described five human neutralizing antibodies from survivors of the 1918 pandemic that bind the hemagglutinin (HA) surface antigen. Here, we define the binding sites of antibodies 1F1 and 1I20 on the 1918 HA and demonstrate that these overlap with the glycan receptor binding site. The glycan specificity differs between human and avian viruses for the linkages of the sialylated sugar receptors [human (α2–6) or avian (α2–3)]. 1F1 and 1I20 binds viruses that contain HA residues that mediate preference for α2–6 sialylated sugars. Three other control antibodies were not affected by preferences for the linkages of the sialylated sugar receptors because they bind elsewhere. Since the receptor-binding site is relatively conserved, this may explain the cross-reactivity of 1F1 and the enhanced binding of 1F1 and 1I20 to HAs with human receptor specificity.

## Introduction

The hemagglutinin (HA) protein of influenza viruses binds to sialic acid receptors on host cells and is the major target of neutralizing antibodies. Amino-acid changes in the immunodominant HA antigenic sites that arise in response to immune selective pressure (antigenic drift) enable seasonal influenza A viruses to cause repeated epidemics and necessitate continuous reevaluation of the composition of influenza vaccines. Characterization of antibodies that display the ability to cross-neutralize divergent viruses may suggest strategies to elicit more broadly protective immunity. The broadest cross-reactive influenza mAbs described to date recognize conserved regions of the HA stem [1], [2], [3], [4], [5], [6] as compared to the HA head region, which is much more variable. Nevertheless, a few cross-reactive antibodies to the HA head have also been found [7], [8], [9], [10], [11], [12], [13], [14]. S139 is a murine monoclonal antibody against antigenic site B [7], but also reaches into the receptor binding site [13]. Recently, human monoclonal antibodies of the V_H_1-69 lineage against the receptor-binding pocket have been described by Ohshima *et al.* [8] and our group [11]. Whittle *et al.* described the H1N1 antibody CH65 [9] which is complementary in its H1N1 activity to our H1N1 antibody 5J8 [10]. Antibody C05 also binds to the receptor binding site of multiple influenza A subtypes using mainly its CDR H3 loop [14]. Recently, a cross-reactive antibody to influenza B CR8033 was shown to bind to the head and overlap with the receptor-binding pocket [12]. Extensive epitope mapping with large panels of murine mAbs previously identified five major antigenic sites on the HA head domain of H1N1 viruses, and these have been termed Sa, Sb (residues 186–198), Ca_1_, Ca_2_ (residues 140–145, 224–225) and Cb [15], [16], [17]. These highly variable surface-exposed regions are located in the membrane-distal end of the HA trimer near the HA RBS.

In a previous study [18], we described five naturally occurring human mAbs that potently inhibit the 1918 H1N1 pandemic influenza virus. The antibodies were cloned from the B cells of individuals born prior to 1918, and were isolated prior to the 2009 H1 pandemic; the mAbs were designated 1F1, 1I20, 2B12, 2D1, and 4D20 [18]. Two of these mAbs, 1F1 and 1I20, independently selected escape mutants of the 1918-like influenza A/Swine/Iowa/15/30 (H1N1) virus with the same proline to histidine escape mutation at HA1 position 186, a residue adjacent to the Sb antigenic site [18]. Sequence analysis with the online antibody database tool IMGT [19] revealed the antibody heavy chain genes encoding mAbs 1F1 and 1I20 used similar V, D, and J gene segments (V_H_3–30, D3–22, J_H_5 for 1F1; V_H_3–30, D3–10, J_H_5 for 1I20; Table S1), but different light chain types (λ for 1F1; κ for 1I20; Table S1). Similar concentrations of 1F1 and 1I20 inhibit the 1918 virus, and administration of either antibody protected mice from lethal 1918 virus challenge [18]. One notable difference between these two antibodies, however, is that mAb 1F1 not only inhibits the 1918 H1N1 virus, but also 1943 as well as select 1947 and 1977 human H1N1 viruses [18]. 1I20 does not inhibit these antigenically drifted post-1918 human viruses.

We used a combination of X-ray crystallography, site-directed mutagenesis, selection of antibody escape mutant viruses and biochemical assays to define the epitopes of 1F1 and the related 1I20 antibodies. The structure of 1F1 in complex with the 1918 HA demonstrated that the 1F1 heavy chain reaches into the HA receptor binding pocket and makes contacts with residues that interact with the sialoglycan receptor. Additional contacts are made with residues from the Sa, Sb and Ca_2_ antigenic sites. Hemagglutination-inhibition and binding assays corroborated the crystal structure data and indicated that the 1I20 mAb likely binds HA in a similar manner as 1F1.

## Results

### Crystal structure of mAb 1F1 in complex with 1918 HA

To better understand the mechanism of 1F1 neutralization and its ability to cross-react with H1N1 viruses separated by decades of virus evolution, we determined the crystal structure of Fab 1F1 in complex with SC1918 HA using diffraction data that extend to 3.3 Å, but we report a nominal resolution of 3.55 Å due to anisotropy (see Table S2). We also determined crystal structures of the 1F1 Fab and “avianized” 1918 HA (SC1918 D190E D225G; AV1918) components separately at high resolution (1.45 Å and 1.80 Å resolution, respectively). The new AV1918 structure was refined at significantly higher resolution than closely related structures reported previously (e.g., SC1918, PDB code 1RUZ) [20], [21]. The availability of these high-resolution models for molecular replacement and as restraints in structure refinement significantly improved the final quality of the lower resolution Fab-HA complex structure. Data collection and refinement statistics are reported in Table S2. The overall structure of AV1918 HA is very similar to previously reported structures for SC1918 HA. The two amino-acid substitutions that differentiate Av1918 from SC1918 (D190E and D225G) had no significant effect on the overall architecture of the receptor binding site, aside from the altered side-chain substitutions.

The crystal structure of the 1F1-SC1918 complex contains two complete HA trimers in the asymmetric unit. Each HA protomer (6 in all for the two trimers) is bound by a single copy of the 1F1 Fab in the expected stoichiometry of 3 Fabs per HA trimer. Of the 6 Fabs in the asymmetric unit, only two are fully ordered. In the remaining four copies, only the variable domains are well defined, as observed in other Fab and Ig-domain containing structures [1], [22], [23]. Together these disordered domains constitute ∼17% of the total expected protein mass in the asymmetric unit. As the variable and constant domains are joined via two flexible hinges in the “elbow” region and these four sets of 1F1 constant domains fail to make significant crystal contacts in the relatively open crystal lattice, these constant regions likely adopt an ensemble of conformations in the crystal and are not interpretable in our electron density maps. Indeed, the Fab elbow angles vary over 30° between the 1F1 Fabs resolved in the free Fab structure (215° and 217°) and in the SC1918 complex (185° and 190°).

**Figure 1:**
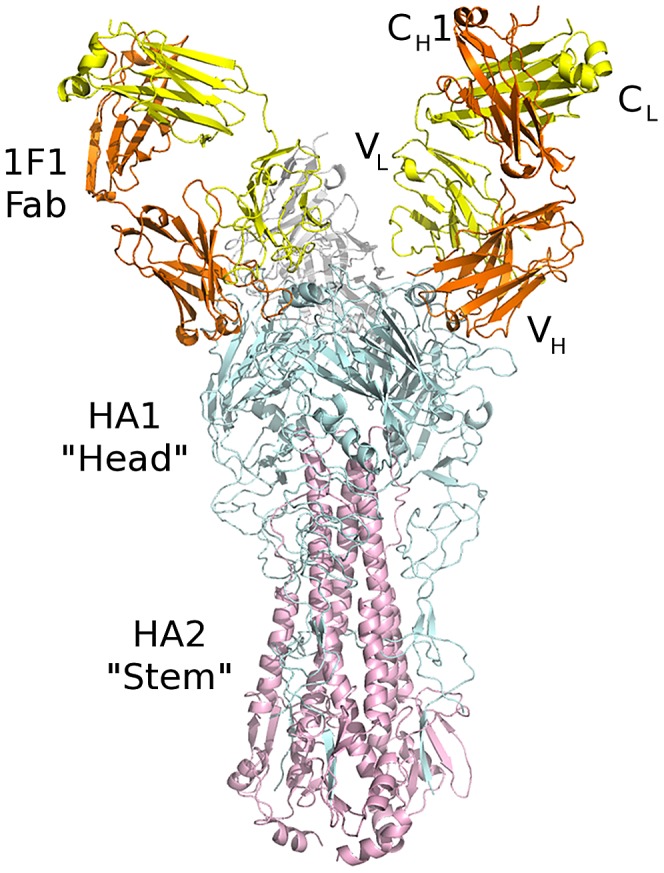
Crystal structure of 1F1-SC1918 complex. Three Fabs bind to an HA trimer. The 1F1 Fab (heavy chain in orange, light chain in yellow) binds to the HA1 “head” subunit (cyan) close to and overlapping the receptor binding site. The HA2 fusion subunit is colored pink. As oriented, the viral membrane would be at bottom and the receptor binding site and target cell would be at the top.

**Figure 2:**
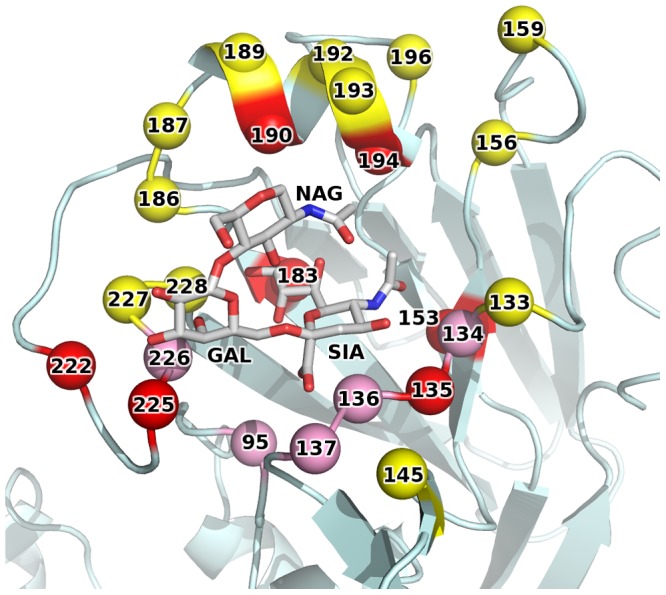
1F1 epitope overlaps the receptor binding site. 1F1 recognizes several residues involved in binding sialic acid receptors (red spheres). Additional residues that contact receptor (but not antibody) are in pink spheres, and additional antibody contact residues are in yellow spheres. A sialic acid receptor is shown to indicate its location, but was not included in the 1F1-HA structure.

Fab 1F1 binds an epitope at the apex of the HA spike, in the HA1 “head” region (Figure 1, overview). The 1F1 epitope contains several residues that typically contact the sialoglycan receptor, including 135, 153, 183, 190, 194, 222, and 225 (Figure 2, RBS with glycan, contact residues labeled). Of particular note are the H1 HA receptor specificity-determining residues 190 and 225 [20], [21], [24], [25], [26], [27]. Many of these contacts are mediated by CDR-H3, which inserts its tip into the RBS. 1F1 also contacts a number of more variable residues outside the RBS, including 133A, 145, 156, 159, 186, 187, 189, 192, 193, 196, 227, and 228 (Figure 2). In total, 1F1 binding to the HA buries a total of 1440 Å^2^ of protein surface at the interface. Of this, approximately 72% is buried by the heavy chain and 28% by the light chain, similar to many other antibodies to proteins.

**Figure 3:**
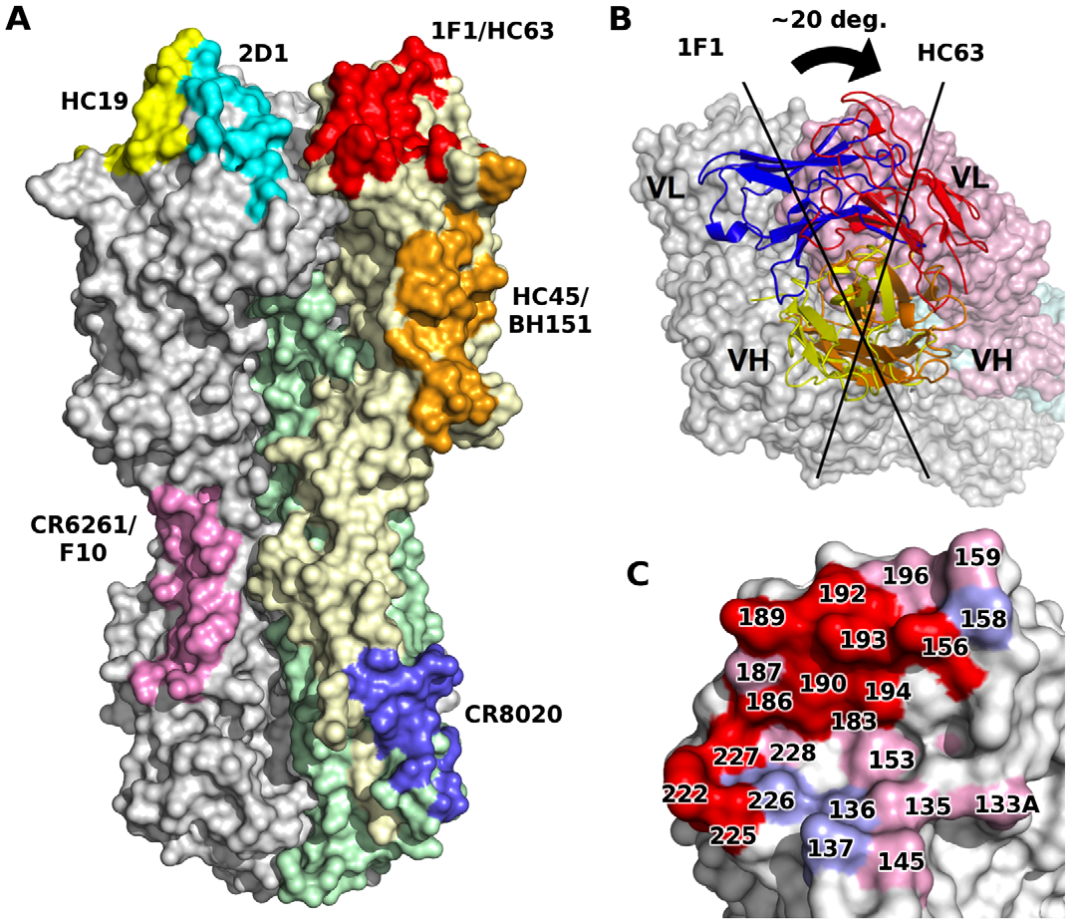
1F1 and HC63 exhibit similar HA binding modes. (A) Comparison of footprints on HA of some representative structurally characterized mAbs. Note that some pairs of antibodies recognize similar epitopes in several different antigenic sites, suggestive of some preferred binding orientations. (B) A rotation about V_H_ results in completely different V_L_ interactions for each 1F1 and HC63 antibody. (C) 1F1 (pink and red) and HC63 (light blue and red) footprints mapped onto HA surface. Overlapping regions are shown in red.

Remarkably, the epitope of 1F1 on H1 HA is similar to the HC63 epitope in H3 HA (PDB code 1KEN) [28], [29], despite highly divergent sequences on both sides of the interface for both the antibodies and the HA targets (Figure 3, 1F1 vs. HC63). Furthermore, the overall orientation of the V_H_ domains from HC63 and 1F1 are very similar, resulting in their CDR-H1, -H2, and -H3 contacting similar surfaces on the H3 and H1 HAs, respectively. In particular, HC63 also inserts the tip of CDR-H3 into the receptor binding site, albeit somewhat less deeply than 1F1 due to a shorter length CDR-H3 (11 residues for HC63 versus 17 for 1F1). However, a slight rotation (∼20°) of the V_H_ domain around its interface with the HA results in significantly different interactions between the light chains of these two antibodies. In contrast to HC63, where its CDR-L1 and -L2 are also centered on the receptor binding site, the 1F1 light chain is displaced by more than 10 Å, moving the tip of CDR-L2 out of the receptor binding site where it binds the outer surface of the 190-helix. Despite these differences, the overall similarity between the HC63 and 1F1 interactions is intriguing, and is suggestive perhaps of a limited number of preferred antibody binding modes, even across subtypes.

### Binding affinity of 1F1 and 1I20 with 1918 wt or avianized variant HAs

Given that the 1F1 epitope is comprised of several residues that typically contact the sialoglycan receptor, 1F1, the related 1I20, and three antibodies, mAbs 2B12, 2D1, and 4D20, that recognize other epitopes, were tested for binding using a label-free biosensor (Table 1) to recombinant, soluble HA proteins possessing SC1918 HA (α2,6SA specificity), NY1918 HA (dual α2,6SA and α2,3SA specificity), or Av1918 HA (α2,3SA specificity). These HAs differ only at positions 190 and 225 [24]. Binding of mAbs 2B12, 2D1, and 4D20, was unaffected by a D190E change, a D225G change converting the South Carolina to the New York sequence, or a double mutant that is Av1918 HA (Table 1). The D190E mutation has reduced affinity for mAb 1F1 by about 250-fold and for 1I20 by about 1,900-fold (Table 1). The D225G mutation led to a reduction of affinity of ∼360-fold for 1F1 and eight-fold for 1I20. Binding of mAb 1I20 to Av1918 was not detected, and a substantially reduced affinity of only 1.0×10^−6^ M was found for mAb 1F1 (Table 1). These data are consistent with the structural data implicating sialoglycan receptor-contacting residues within the 1F1 epitope. 1I20 mAb bound to a similar epitope consistent with the fact that it shares common genetic elements with 1F1 [30], although both antibodies are derived from different clonal ancestors because of different junctions, different light chains, and a different pedigree of somatic mutations.

**Table 1:** Binding affinities of five human mAbs for 1918*wt* HA or its avianized variants.

Virus source of HA (Wild-type or A/SC/1/18 variant)	K_D_ of binding [M] of 1918 antibody Fab with 1918 and variant HAs			
	1F1	1I20	2B12	2D1
1918*wt*	6.2×10^−9^	3.3×10^−9^	9.8×10^−9^	2.5×10^−9^
D190E mutant	2.4×10^−7^	1.7×10^−6^	1.2×10^−8^	5.8×10^−9^
D225G mutant (NY1918)	1.7×10^−7^	3.9×10^−8^	1.3×10^−8^	6.2×10^−9^
D190E/D225G mutant (Av1918)	1.0×10^−6^	<	2.1×10^−8^	3.1×10^−9^

“<” Denotes no detectable binding, with an estimated minimal detectable binding of ∼1×10^−6^ M.

### HA escape mutations selected by mAb 1F1

**Figure 4:**
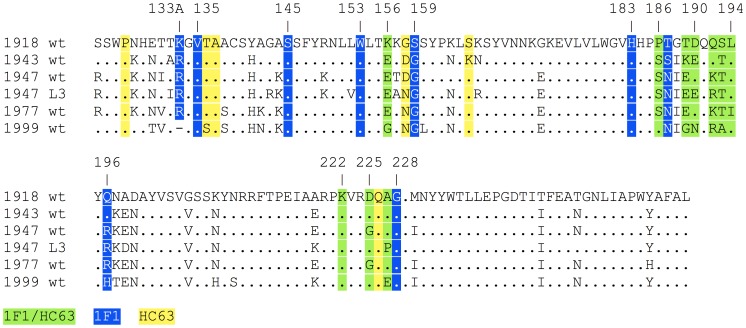
1F1 and HC63 contacts on HA. HA residues contacted by both 1F1 and HC63 (green), 1F1 only (blue), or HC63 only (yellow) mapped onto a structure-based sequence alignment of H1 HAs bound by 1F1 (1918, 1943, 1947 L3, 1947 FM, 1977, and 1999). “.” indicates identity with the 1918 *wt* sequence.

**Figure 5:**
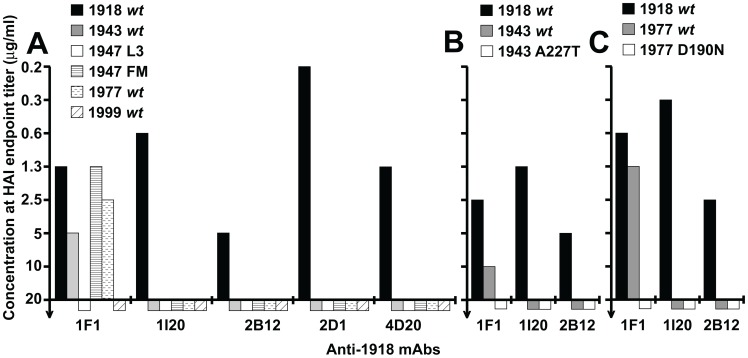
Role of HA residues 190 and 227 in neutralization of 1943 and 1977 H1N1 viruses, respectively. (A) HAI assays performed with mAbs 1F1, 1I20, 2B12, 2D1, or 4D20 against VLPs based on *wt* viruses indicated in Figure 1. The maximum concentration of antibody tested in each assay was 20 µg/ml. (B) HAI assays performed with mAbs 1I20, 1F1 and 2B12 against VLPs possessing the following HAs: 1918 *wt*; 1943 *wt*; 43A227T (1943 HA with mutation A227T). (C) HAI assays performed with mAbs 1I20, 1F1 and 2B12 against VLPs possessing the following HAs: 1918 *wt*; 1977 *wt*; 77D190N (1977 HA with residue D190 mutated to N).

Although influenza A/South Carolina/1/18, A/Weiss/43, A/Fort Monmouth/1/47, and A/USSR/92/77 (referred to below as the 1918, 1943, 1947 FM, and 1977 viruses, respectively) possess divergent HAs, including divergent Sb sites (Figure 4), mAb 1F1 inhibits hemagglutination by each of these viruses (Figure 5A) [18]. To map HA residues critical for HAI activity against the 1943 and 1977 viruses, 1F1 escape mutants for 1943 and 1977 viruses were selected. Sequencing of escape mutant HA genes of 1943 virus escape mutants reproducibly identified an A227T mutation in HA1. Sequences derived from 1977 escape mutant viruses possessed a D190N change in HA1, although some escape mutant strains appeared to be mixed populations that also contained an S186F change. To confirm that individual changes at positions 190 and 227 are sufficient to confer escape from inhibition, we introduced the changes into cDNAs encoding the HAs of 1943 or 1977 H1N1 viruses and produced virus-like particles (VLPs). As expected, 1F1 lost HAI activity towards the A227T 1943 HA (Figure 5B) and the D190N mutant 1977 HA (Figure 5C).

**Figure 6:**
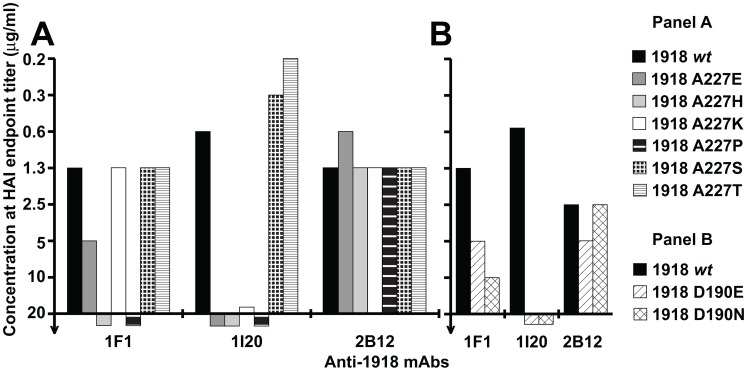
Role of HA residues 186, 190 and 227 for 1F1 and 1I20 interaction with the 1918 HA. (A) HAI assays performed with mAbs 1I20, 1F1 and 2B12 against VLPs possessing 1918 HAs corresponding to the wild-type sequence (1918 *wt*) or with HAs containing the indicated point mutations at residue 227. (B) HAI assays performed with mAbs 1I20, 1F1 and 2B12 against VLPs possessing 1918 HAs corresponding to the wild-type sequence (1918 *wt*) or HAs with the indicated point mutations at residue 190.

Prior studies to select for 1F1 and 1I20 escape mutants in a virus closely related to the 1918 virus, influenza A/swine/Iowa/30 (H1N1), repeatedly resulted in changes at residue 186 [18]. To determine whether the residues identified by escape mutant selection for the 1943 and 1977 viruses could also be implicated in 1F1 HAI activity towards the 1918 HA, residues 190 and 227 were mutated in the context of the 1918 HA. When an A227T mutation was introduced into a 1918 virus HA, it did not affect 1F1 HAI activity. However, alignment of the HAs of 20^th^ century human H1N1 isolates identified several other amino acids that have appeared at position 227 (Figures 4, 6A). When these were introduced into the 1918 HA, two mutations, A227H and A227P, substantially impacted 1F1 HAI activity, demonstrating a role for residue 227 in inhibition of 1918 HA by both 1F1 and 1I20 (Figure 6A). When a D190N mutation was introduced into a 1918 HA, 1F1 HAI activity was partially abrogated (Figure 6B). These data support a role for residues 190 and 227 in 1F1 inhibition of the 1918 virus as well as in the 1943 and 1977 viruses.

### Structural interpretation of escape mutant data

The three HA residues involved in the four escape mutations selected by 1F1 (P186H, S186F, D190N, and A227T) all map to the HA-1F1 interface in the crystal structure. The P186H and S186F mutations introduce larger side chains at a position buried in the interface and likely lead to a steric clash between the HA and CDR-H3. It is less clear how the D190N and A227T mutations led to virus escape, as they did not completely abolish 1F1 HAI activity when introduced in the 1918 HA as compared to 1977 HA. Given the somewhat conservative nature of these substitutions, they may be expected to have a small effect on the binding of 1F1 for HA, which is insufficient to reduce antibody binding below the threshold necessary for escape. However, in the context of HAs bound by 1F1 with lower affinity, such as 1943 and 1977, these more subtle mutations may reduce 1F1 binding beyond what is required for effective neutralization.

## Discussion

Relatively few mAbs that bind the influenza virus HA head have been demonstrated to neutralize significantly divergent strains [7], [8], [9], [10], [11]. A greater understanding of the basis for such broadly cross-reactive antibodies may suggest novel vaccine or therapeutic approaches to influenza virus infection. This study provides a detailed characterization of one such antibody, 1F1, and highlights common, emerging properties of cross-reactive anti-head antibodies. Notably, such antibodies appear to react with the relatively conserved receptor binding domain, reach into the receptor binding pocket and/or are sensitive to changes in HA receptor binding specificity [7], [8], [9], [10], [11], [12], [13], [14]. The availability of 1I20, a mAb that shares a heavy chain closely related to that of 1F1, but did not neutralize the 1943, 1947 or 1977 viruses tested, provides additional insight. Because 1I20 maps to the same epitope as 1F1, it is very likely that 1I20 will also reach into the receptor binding pocket. This finding suggests that not all mAbs that make contact with the RBS will be broadly cross-reactive and that additional determinants of cross-reactivity exist as the antibody footprint typically includes surrounding hypervariable loops as well.

We previously characterized 1F1 and 1I20 as Sb site antibodies based on the P186H escape mutation for a residue that is immediately adjacent to the Sb site (residues 187–197 [16]). However, this antigenic site definition is based on BALB/c mouse hybridoma antibodies against influenza A/Puerto Rico/8/1934 (H1N1) [15], [16], [17]. Those mapping studies, while extensive, cannot necessarily be considered complete especially as applied to the human antibody response [15], [17]. Also, co-crystal structures of H1 HA antibodies binding the globular head that would validate the conventional definition for H1 HA are limited. We have shown previously that the epitope of the H1 HA Sa site antibody 2D1 extends to residues beyond the conventionally defined antigenic site to sites Sb and Ca_1_ [31]. Here, the crystal structure demonstrates that 1F1 interacts with residues within Sa, Sb and Ca_2_ and also reaches into the HA receptor binding pocket. This epitope is strikingly similar to that described for HC63 [29], an H3-specific mouse mAb that also exhibited HAI activity against multiple divergent H3N2 viruses [28]. Only a limited number of H1N1 antibodies have been crystallized in complex with their respective HAs. Their epitopes seem to be comprised of multiple antigenic sites, rather than just a single distinct antigenic site. It might now be time to get away from the original classification of epitope sites. Still, this conventional definition remains useful for ease of topographic orientation on the HA head. HC63 appears to neutralize virus through two distinct mechanisms: The direct occlusion of the receptor binding site by V_H_ interferes with receptor binding and virus attachment [29]. Interestingly, HC63 inhibits the pH-induced conformational changes associated with membrane fusion [29], presumably by cross-linking or otherwise interfering with the separation of the heads, which has been suggested to be required to allow efficient reorganization of HA2 to its fusion-active conformation [32]. In contrast, while 1F1 potently inhibits receptor binding, it was unable to block the pH-induced conformational changes in HA that drive membrane fusion (data not shown). While the overall binding modes of 1F1 and HC63 are similar, the HC63 footprint extends across the interface between two adjacent HA1 subunits [29], while that of 1F1 is wholly contained within a single HA1 domain and cannot cross-link the subunits of the trimer as a monovalent Fab.

The extension of the 1F1/1I20 epitope towards the RBS and the modest conservation of the residues in this pocket may explain in part why 1F1 shows cross-reactivity towards later 1943, 1947, and 1977 viruses. The related mAb 1I20 does not inhibit these viruses, likely as a result of numerous substitutions in contact positions in CDR-H1 and -H3 (Table S1). In contrast, mAbs 2B12, 2D1, and 4D20 are not affected by changes in human versus avian receptor specificity since their epitopes do not involve those residues.

The HA receptor specificity of influenza A virus strains is a determinant of virus transmissibility and virulence. Therefore, methods to rapidly and easily determine HA receptor specificity would be of interest for influenza virus surveillance and research purposes. 1F1 and other antibodies with broad reactivity and which are sensitive to changes in receptor specificity could serve as such reagents. The conserved receptor-binding pocket may also be an attractive target for universal or improved influenza vaccine design as a complement to targeting the hemagglutinin stem. Structures like those of the 1F1-HA complex may serve as templates for such vaccine constructs.

## Materials and Methods

### Nomenclature

For the experiments described below, H1N1 HA influenza sequences are used based on the following strains (abbreviation in brackets): A/South Carolina/1/18 (1918 *wt*), A/Weiss/43 (1943 *wt*), A/Fort Monmouth/1/47 (1947 FM), A/USA/L3/47 (1947 L3) [33] (GenBank accession number GI: 343409202), A/USSR/92/77 (1977 *wt*), and A/New Caledonia/20/99 (1999 *wt*) virus. The stated positions of all HA residues designated in this manuscript are based on the amino-acid numbering conventions used for H3 [34].

### Antibody protein expression and purification

The antibody proteins were expressed recombinantly in mammalian cells as described [31]. Briefly, we cloned the matched heavy or light chain gene by RT-PCR (mAb 1F1 heavy/λ, mAb 2B12 heavy/λ, mAb 2D1 λ, mAb 4D20 λ) using In-Fusion enzyme (Clontech/Takara Bio) into opened pEE6.4 or pEE12.4 vectors (Lonza Group Ltd), respectively. These vectors were modified to contain mouse κ leader sequences. cDNA of the remaining antibody chains was synthesized (GeneArt) based on the published nucleotide sequences and cloned into the expression vectors. 1F1 Fab was produced by limited proteolysis of 1F1 IgG by endoprotease Lys-C. Digests containing ∼0.25 µg Lys-C per 1 mg 1F1 IgG in 25 mM Tris, 1 mM EDTA, pH 8.5 were incubated at 37°C for 4 hours, then stopped by the addition of TLCK and leupeptin to a final concentration of ∼1 mM and 0.4 mM, respectively. Alternatively, Fabs were expressed recombinantly by introducing a stop codon into the heavy chain gene immediately after the codon for the cysteine of the hinge disulfide. DH5α cells were transformed with plasmids for EndoFree Maxi preparation (Qiagen). Purified DNA was co-transfected transiently into HEK 293F cells (Invitrogen) using PolyFect reagent (Qiagen) in disposable shaker flasks. The supernatant was harvested on day seven and purified through a gravity column with CaptureSelect Fab λ resin (BAC B.V., GP Naarden, The Netherlands) in D-PBS for Fabs 1F1, 2B12, 2D1, and 4D20 or purified on an ÅKTA FPLC instrument (GE Healthcare Life Sciences) using HiTrap Protein G columns (GE; all other proteins) and concentrated with 15 mL centrifugal filter units with 30 kD molecular weight cut-off (Millipore, Billerica, MA). The purity of all expressed proteins was assessed using reducing, denaturing SDS-PAGE gel electrophoresis (Invitrogen).

### Expression, purification and crystallization of 1F1 Fab, Av1918 HA, and the 1F1-SC1918 complex

The SC1918 and Av1918 HAs were expressed using the baculovirus system and purified essentially as previously described [1]. Following initial capture of 1F1 Fab from Lys-C digest of IgG (Protein G affinity chromatography) or from cell culture supernatant from recombinant expression (a-lambda), Fab was further purified by cation exchange chromatography (MonoS, GE Healthcare) in sodium acetate buffer, pH 5.0 and a linear NaCl gradient from 0–1 M. Fractions containing Fab were buffer exchanged into 10 mM Tris, pH 8.0, 150 mM NaCl and subjected to gel filtration (Superdex 200, GE Healthcare). Purified SC1918 HA was mixed with a stoichiometric excess of recombinant 1F1 Fab and the complex was isolated from free Fab by gel filtration.

Initial crystallization screening for 1F1 Fab (14 mg/mL), Av1918 HA (28 mg/mL), and the 1F1-SC1918 complex (9.6 mg/mL) was conducted using the robotic CrystalMation system (Rigaku) at the Joint Center for Structural Genomics (JCSG; www.jcsg.org). Diffraction quality crystals were subsequently grown in sitting drops by vapor diffusion (0.5 µL protein solution +0.5 µL well solution with 1 mL reservoir for 1F1 Fab and 1F1-SC1918 complex; 100 nL protein solution +100 nL well solution with 200 µL reservoir for Av1918 HA). Crystals used for data collection were grown at 20°C from 20% PEG 4000, 200 mM dibasic sodium phosphate (1F1 Fab); 4°C from 100 mM Tris pH 8.0, 40% MPD (2-methyl-2,4-pentanediol (Av1918); or 20°C from 8.5% PEG 6000, 100 mM Tris pH 7.9 (1F1-SC1918 complex). Crystals were cryprotected in the mother liquor (Av1918), with well solution supplemented with 15% glycerol (1F1 Fab), or with 25% ethylene glycol (1F1-SC1918 complex) and flash cooled in liquid nitrogen.

Diffraction data were collected on the General Medicine/Cancer Institutes Collaborative Access Team (GM/CA-CAT) beamline 23ID-D at the Advanced Photon Source at Argonne National Laboratory (Av1918) and on beamline 11-1 (1F1 Fab and 1F1-SC1918 Complex) at the Stanford Synchrotron Radiation Lightsource (SSRL). The data were indexed integrated and scaled using HKL2000 (HKL Research), and merged using Xprep (Bruker). The structures were solved by molecular replacement using Phaser [35]. Structures from PDB codes 2FB4 and 1RZF (1F1 Fab, variable domains and constant domains, respectively); 1RUZ (Av1918), or the 1F1 Fab and Av1918 coordinates reported here (1F1-SC1918 complex), were used as search models and total of 2 Fabs (1F1 Fab), 1 HA trimer (3 protomers) (Av1918), or two HA trimers (six protomers), 2 Fabs, and 4 sets of V_H_/V_λ_ domains (1F1-SC1918 complex) were ordered in the asymmetric unit. In the 1F1-SC1918 complex, attempts to place the remaining 4 sets of C_H_1/C_λ_ domains were unsuccessful, and very little density was observed for the missing protein components after refinement, suggesting that these domains are disordered in the crystal. As V_H_ and V_λ_ are joined to C_H_1 and C_λ_ by flexible linkers, the relative orientations of the variable and constant domains are not rigidly defined and, in the absence of stabilizing crystal contacts, the constant domains can likely adopt an ensemble of conformations in the relatively open crystal lattice. Rigid body refinement, simulated annealing and restrained refinement (including TLS refinement) were carried out in Phenix [36]. Riding hydrogens were used during refinement. Between rounds of refinement, the model was built and adjusted using Coot [37]. Waters were built automatically into the 1F1 Fab and Av1918 HA models using the “ordered_solvent” modeling function in Phenix [36]. Refinement statistics can be found in Table S2. A depiction of the representative electron density at the 1F1-HA interface can be found in Figure S3.

The coordinates and structure factors for 1F1 Fab, Av1918 HA, and the 1F1-SC1918 complex have been deposited in the Protein Data Bank (PDB) with accession numbers 4GXV, 4GXX, and 4GXU, respectively.

### VLP expression and HAI assays

Expression plasmids encoding the parental or mutated 1918, 1943 or 1977 HA proteins were co-expressed with an N1 neuraminidase to produce VLPs in 293T cells, as described previously [18], [38]. Briefly, VLP were generated by co-transfection of 293T cells with 1 µg each of expression plasmids for HA and NA. Two days post-transfection, supernatants were collected. HAI assays were performed as described [39]. Briefly, serially diluted antibodies were pre-incubated with 8 hemagglutinating units of virus or VLP per well. Chicken red blood cells were added to a final concentration of 0.5% and the plate was incubated on ice for 30–60 min.

### Isolation and characterization of antibody escape mutant viruses

Antibody escape mutant influenza A/Weiss/43 and A/USSR/92/77 viruses were selected [15], [40]. Briefly, escape mutant viruses were selected by treatment of virus with excess antibody, followed by recovery of inhibition resistant viruses in 10-day-old embryonated chicken eggs. RNA was extracted from virus-infected allantoic fluid, then cDNA was generated by RT-PCR, directly cloned, sequenced, and aligned to previously determined *wt* virus HA gene sequences.

### Biosensor studies to determine affinity

Binding affinity of recombinant 1918 Fabs to recombinant trimerized His-tagged HA protein containing the sequence of 1918 *wt* or its avianized variant strains was measured using anti-Penta-HIS tips on the Octet QK platform (FortéBio, Menlo Park, CA). The soluble HA protein was expressed and purified as described [41]. Data were calculated using Origin 7.5 SR6 software (OriginLab Corp., Northampton, MA) based on automated curve fittings prompted by the Octet 4 software (ForteBio) using a 1∶1 binding model. All measurements are listed as the average of two duplicate measurements. The Fabs were diluted to a concentration of 60 µg/mL (1F1, 1I20, 2B12) or 30 µg/mL (2D1, 4D20). Curve fittings and experimental errors can be found in the supporting information (Figure S2, Table S3).

## Supporting Information

Figure S1 HAI assays performed with mAbs 1F1 and 1I20 against VLPs containing 1918 HAs with wild-type sequence (wt) or the indicated point mutations.
(PDF)

Figure S2 Automated curve fittings prompted by the Octet 4 software (ForteBio) of Fab 1F1, 1I20, 2B12, 2D1, or 4D20 affinities in association with the wild type SC1918 HA, a D190E variant, the D225G variant (NY1918), or the D190E/D225G double mutant (AV1918).
(PDF)

Figure S3 Representative electron density at the 1F1-HA interface, in the vicinity of CDR H3. The antibody and HA are depicted as magenta and yellow sticks, respectively. The 2F_O_-F_C_ electron density map (blue mesh) is contoured at 1 sigma.
(TIF)

Table S1 Sequence alignment of 1F1 and 1I20 heavy chains (GenBank GI:309753504 and GI:163931316, respectively). The amino-acid residue numbering follows the Kabat scheme to be consistent with the numbering in other crystal structures in the PDB. The framework/loop definitions are based on the international ImMunoGeneTics information system (IMGT).
(PDF)

Table S2 Data collection and refinement statistics.
(PDF)

Table S3 Affinity measurement data of Fabs 1F1, 1I20, 2B12, 2D1, or 4D20 in association with the wild type SC1918 HA, a D190E variant, the D225G variant (NY1918), or the D190E/D225G double mutant (AV1918).
(PDF)

Table S4 1F1-HA contacts. Summary of interacting residue pairs from chains A, B, M, and N in the 1F1-Sc1918 crystal structure, generated using CONTACSYM [42].
(PDF)

## References

[1] Ekiert DC, Bhabha G, Elsliger MA, Friesen RH, Jongeneelen M, et al (2009) Antibody recognition of a highly conserved influenza virus epitope. Science 324: 246–251. 19251591 10.1126/science.1171491 PMC2758658

[2] Kashyap AK, Steel J, Oner AF, Dillon MA, Swale RE, et al (2008) Combinatorial antibody libraries from survivors of the Turkish H5N1 avian influenza outbreak reveal virus neutralization strategies. Proc Natl Acad Sci U S A 105: 5986–5991. 18413603 10.1073/pnas.0801367105 PMC2329690

[3] Okuno Y, Isegawa Y, Sasao F, Ueda S (1993) A common neutralizing epitope conserved between the hemagglutinins of influenza A virus H1 and H2 strains. J Virol 67: 2552–2558. 7682624 10.1128/jvi.67.5.2552-2558.1993 PMC237575

[4] Sui J, Hwang WC, Perez S, Wei G, Aird D, et al (2009) Structural and functional bases for broad-spectrum neutralization of avian and human influenza A viruses. Nat Struct Mol Biol 16: 265–273. 19234466 10.1038/nsmb.1566 PMC2692245

[5] Throsby M, van den Brink E, Jongeneelen M, Poon LL, Alard P, et al (2008) Heterosubtypic neutralizing monoclonal antibodies cross-protective against H5N1 and H1N1 recovered from human IgM+ memory B cells. PLoS One 3: e3942. 19079604 10.1371/journal.pone.0003942 PMC2596486

[6] Wrammert J, Koutsonanos D, Li GM, Edupuganti S, Sui J, et al (2011) Broadly cross-reactive antibodies dominate the human B cell response against 2009 pandemic H1N1 influenza virus infection. J Exp Med 208: 181–193. 21220454 10.1084/jem.20101352 PMC3023136

[7] Yoshida R, Igarashi M, Ozaki H, Kishida N, Tomabechi D, et al (2009) Cross-protective potential of a novel monoclonal antibody directed against antigenic site B of the hemagglutinin of influenza A viruses. PLoS Pathog 5: e1000350. 19300497 10.1371/journal.ppat.1000350 PMC2652660

[8] Ohshima N, Iba Y, Kubota-Koketsu R, Asano Y, Okuno Y, et al (2011) Naturally occurring antibodies in humans can neutralize a variety of influenza virus strains, including H3, H1, H2, and H5. J Virol 85: 11048–11057. 21865387 10.1128/JVI.05397-11 PMC3194982

[9] Whittle JR, Zhang R, Khurana S, King LR, Manischewitz J, et al (2011) Broadly neutralizing human antibody that recognizes the receptor-binding pocket of influenza virus hemagglutinin. Proc Natl Acad Sci U S A 108: 14216–14221. 21825125 10.1073/pnas.1111497108 PMC3161572

[10] Krause JC, Tsibane T, Tumpey TM, Huffman CJ, Basler CF, et al (2011) A broadly neutralizing human monoclonal antibody that recognizes a conserved, novel epitope on the globular head of the influenza H1N1 virus hemagglutinin. J Virol 85: 10905–10908. 21849447 10.1128/JVI.00700-11 PMC3187471

[11] Krause JC, Tsibane T, Tumpey TM, Huffman CJ, Albrecht R, et al (2012) Human monoclonal antibodies to pandemic 1957 H2N2 and pandemic 1968 H3N2 influenza viruses. J Virol 86: 6334–6340. 22457520 10.1128/JVI.07158-11 PMC3372199

[12] Dreyfus C, Laursen NS, Kwaks T, Zuijdgeest D, Khayat R, et al (2012) Highly conserved protective epitopes on influenza B viruses. Science 337: 1343–1348. 22878502 10.1126/science.1222908 PMC3538841

[13] Lee PS, Yoshida R, Ekiert DC, Sakai N, Suzuki Y, et al (2012) Heterosubtypic antibody recognition of the influenza virus hemagglutinin receptor-binding site enhanced by avidity. Proc Natl Acad Sci U S A 109: 17040–17045. 23027945 10.1073/pnas.1212371109 PMC3479480

[14] Ekiert DC, Kashyap AK, Steel J, Rubrum A, Bhabha G, et al (2012) Cross-neutralization of influenza A viruses mediated by a single antibody loop. Nature 489: 526–532. 22982990 10.1038/nature11414 PMC3538848

[15] Caton AJ, Brownlee GG, Yewdell JW, Gerhard W (1982) The antigenic structure of the influenza virus A/PR/8/34 hemagglutinin (H1 subtype). Cell 31: 417–427. 6186384 10.1016/0092-8674(82)90135-0

[16] Brownlee GG, Fodor E (2001) The predicted antigenicity of the haemagglutinin of the 1918 Spanish influenza pandemic suggests an avian origin. Philos Trans R Soc Lond B Biol Sci 356: 1871–1876. 11779386 10.1098/rstb.2001.1001 PMC1088563

[17] Gerhard W, Yewdell J, Frankel M, Webster R (1981) Antigenic structure of influenza virus haemagglutinin defined by hybridoma antibodies. Nature 290: 713–717. 6163993 10.1038/290713a0

[18] Yu X, Tsibane T, McGraw PA, House FS, Keefer CJ, et al (2008) Neutralizing antibodies derived from the B cells of 1918 influenza pandemic survivors. Nature 455: 532–536. 18716625 10.1038/nature07231 PMC2848880

[19] Lefranc MP, Giudicelli V, Ginestoux C, Jabado-Michaloud J, Folch G, et al (2009) IMGT, the international ImMunoGeneTics information system. Nucleic Acids Res 37: D1006–1012. 18978023 10.1093/nar/gkn838 PMC2686541

[20] Gamblin S, Haire L, Russell R, Stevens D, Xiao B, et al (2004) The structure and receptor binding properties of the 1918 influenza hemagglutinin. Science 303: 1838–1842. 14764886 10.1126/science.1093155

[21] Stevens J, Corper AL, Basler CF, Taubenberger JK, Palese P, et al (2004) Structure of the uncleaved human H1 hemagglutinin from the extinct 1918 influenza virus. Science 303: 1866–1870. 14764887 10.1126/science.1093373

[22] Verdino P, Witherden DA, Havran WL, Wilson IA (2010) The molecular interaction of CAR and JAML recruits the central cell signal transducer PI3K. Science 329: 1210–1214. 20813955 10.1126/science.1187996 PMC2951132

[23] Colman PM, Tulip WR, Varghese JN, Tulloch PA, Baker AT, et al (1989) Three-dimensional structures of influenza virus neuraminidase-antibody complexes. Philos Trans R Soc Lond B Biol Sci 323: 511–518. 2569208 10.1098/rstb.1989.0028

[24] Glaser L, Stevens J, Zamarin D, Wilson IA, Garcia-Sastre A, et al (2005) A single amino acid substitution in 1918 influenza virus hemagglutinin changes receptor binding specificity. J Virol 79: 11533–11536. 16103207 10.1128/JVI.79.17.11533-11536.2005 PMC1193621

[25] Stevens J, Blixt O, Glaser L, Taubenberger JK, Palese P, et al (2006) Glycan microarray analysis of the hemagglutinins from modern and pandemic influenza viruses reveals different receptor specificities. J Mol Biol 355: 1143–1155. 16343533 10.1016/j.jmb.2005.11.002

[26] Connor RJ, Kawaoka Y, Webster RG, Paulson JC (1994) Receptor specificity in human, avian, and equine H2 and H3 influenza virus isolates. Virology 205: 17–23. 7975212 10.1006/viro.1994.1615

[27] Naeve CW, Hinshaw VS, Webster RG (1984) Mutations in the hemagglutinin receptor-binding site can change the biological properties of an influenza virus. J Virol 51: 567–569. 6748165 10.1128/jvi.51.2.567-569.1984 PMC254476

[28] Daniels PS, Jeffries S, Yates P, Schild GC, Rogers GN, et al (1987) The receptor-binding and membrane-fusion properties of influenza virus variants selected using anti-haemagglutinin monoclonal antibodies. The EMBO Journal 6: 1459–1465. 3608984 10.1002/j.1460-2075.1987.tb02387.x PMC553952

[29] Barbey-Martin C, Gigant B, Bizebard T, Calder LJ, Wharton SA, et al (2002) An antibody that prevents the hemagglutinin low pH fusogenic transition. Virology 294: 70–74. 11886266 10.1006/viro.2001.1320

[30] Krause JC, Tsibane T, Tumpey TM, Huffman CJ, Briney BS, et al (2011) Epitope-specific human influenza antibody repertoires diversify by B cell intraclonal sequence divergence and interclonal convergence. Journal of Immunology 187: 3704–3711. 10.4049/jimmunol.1101823 PMC3178754 21880983

[31] Xu R, Ekiert D, Krause J, Hai R, Crowe JJ, et al (2010) Structural basis of preexisting immunity to the 2009 H1N1 pandemic influenza virus. Science 328: 357–360. 20339031 10.1126/science.1186430 PMC2897825

[32] Kemble GW, Bodian DL, Rose J, Wilson IA, White JM (1992) Intermonomer disulfide bonds impair the fusion activity of influenza virus hemagglutinin. Journal of Virology 66: 4940–4950. 1629960 10.1128/jvi.66.8.4940-4950.1992 PMC241339

[33] Hirst GK (1952) Strain-specific elements in influenza antigens. J Exp Med 96: 589–603. 13022853 10.1084/jem.96.6.589 PMC2136176

[34] Wilson IA, Skehel JJ, Wiley DC (1981) Structure of the haemagglutinin membrane glycoprotein of influenza virus at 3 A resolution. Nature 289: 366–373. 7464906 10.1038/289366a0

[35] McCoy AJ, Grosse-Kunstleve RW, Adams PD, Winn MD, Storoni LC, et al (2007) Phaser crystallographic software. Journal of Applied Crystallography 40: 658–674. 19461840 10.1107/S0021889807021206 PMC2483472

[36] Adams PD, Afonine PV, Bunkoczi G, Chen VB, Davis IW, et al (2010) PHENIX: a comprehensive Python-based system for macromolecular structure solution. Acta crystallographica Section D, Biological Crystallography 66: 213–221. 20124702 10.1107/S0907444909052925 PMC2815670

[37] Emsley P, Lohkamp B, Scott WG, Cowtan K (2010) Features and development of Coot. Acta Crystallogr D Biol Crystallogr 66: 486–501. 20383002 10.1107/S0907444910007493 PMC2852313

[38] Chen BJ, Leser GP, Morita E, Lamb RA (2007) Influenza virus hemagglutinin and neuraminidase, but not the matrix protein, are required for assembly and budding of plasmid-derived virus-like particles. J Virol 81: 7111–7123. 17475660 10.1128/JVI.00361-07 PMC1933269

[39] Kendal AP, Skehel JJ, Pereira MS (1982) World Health Organization Collaborating Centers for Reference and Research on Influenza: concepts and procedures for laboratory-based influenza surveillance. Atlanta, GA: World Health Organization Collaborating Centers for Reference and Research in Influenza, Centers for Disease Control. B17–B35 p.

[40] Yewdell JW, Webster RG, Gerhard WU (1979) Antigenic variation in three distinct determinants of an influenza type A haemagglutinin molecule. Nature 279: 246–248. 86955 10.1038/279246a0

[41] Krause JC, Tumpey TM, Huffman CJ, McGraw PA, Pearce MB, et al (2010) Naturally occurring human monoclonal antibodies neutralize both 1918 and 2009 pandemic influenza A (H1N1) viruses. J Virol 84: 3127–3130. 20042511 10.1128/JVI.02184-09 PMC2826039

[42] Sheriff S, Hendrickson WA, Smith JL (1987) Structure of myohemerythrin in the azidomet state at 1.7/1.3 A resolution. Journal of Molecular Biology 197: 273–296. 3681996 10.1016/0022-2836(87)90124-0

