# Supplementary figures and images for: Influenza Human Monoclonal Antibody 1F1 Interacts with Three Major Antigenic Sites and Residues Mediating Human Receptor Specificity in H1N1 Viruses
Source: PLoS Pathog. 2012 Dec 6;8(12):e1003067. doi: 10.1371/journal.ppat.1003067 (PMC3516549; doi:10.1371/journal.ppat.1003067)

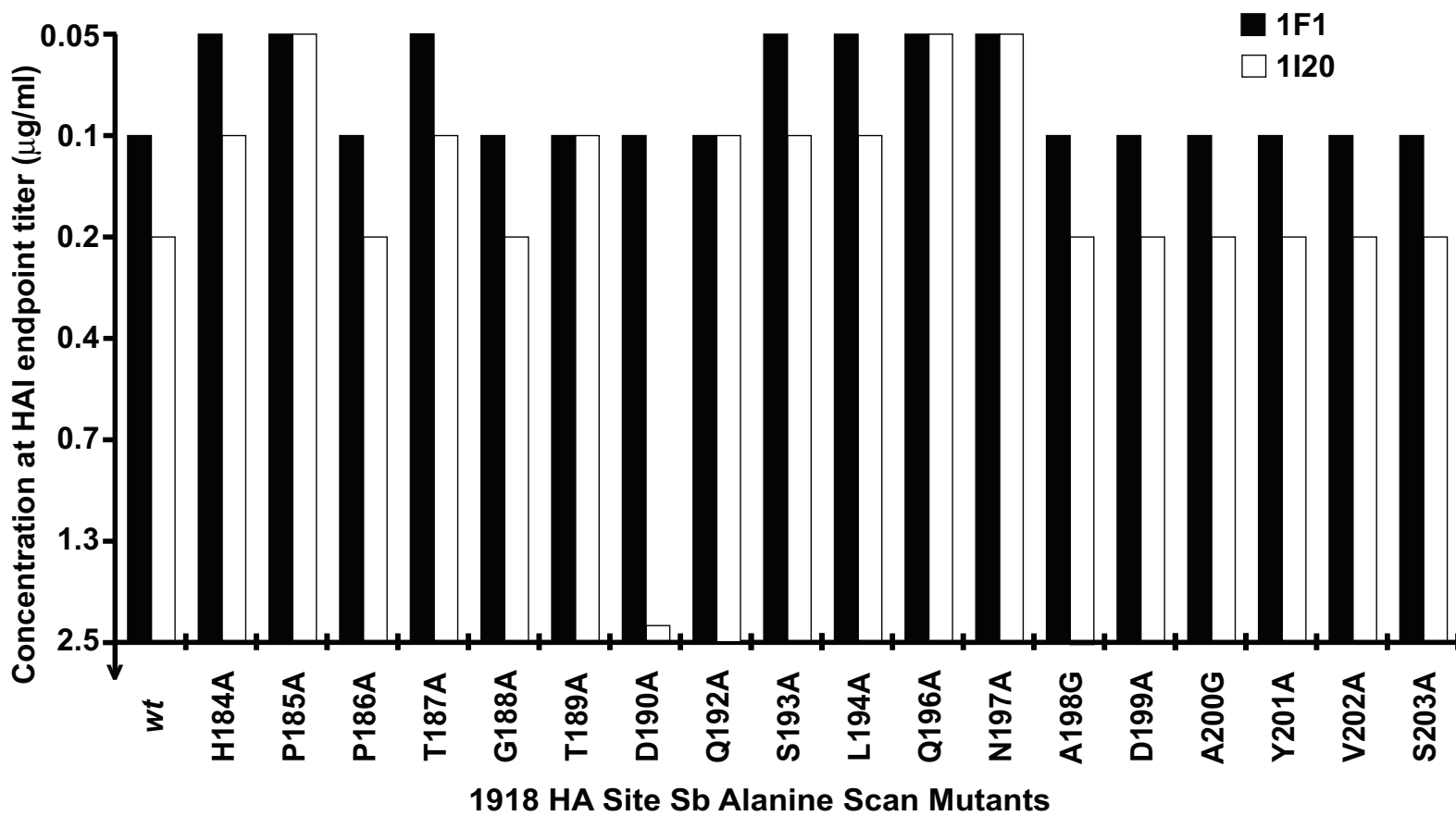

Supplement: Figure S1 — HAI assays performed with mAbs 1F1 and 1I20 against VLPs containing 1918 HAs with wild-type sequence (wt) or the indicated point mutations. (PDF) [file ppat.1003067.s001.pdf]

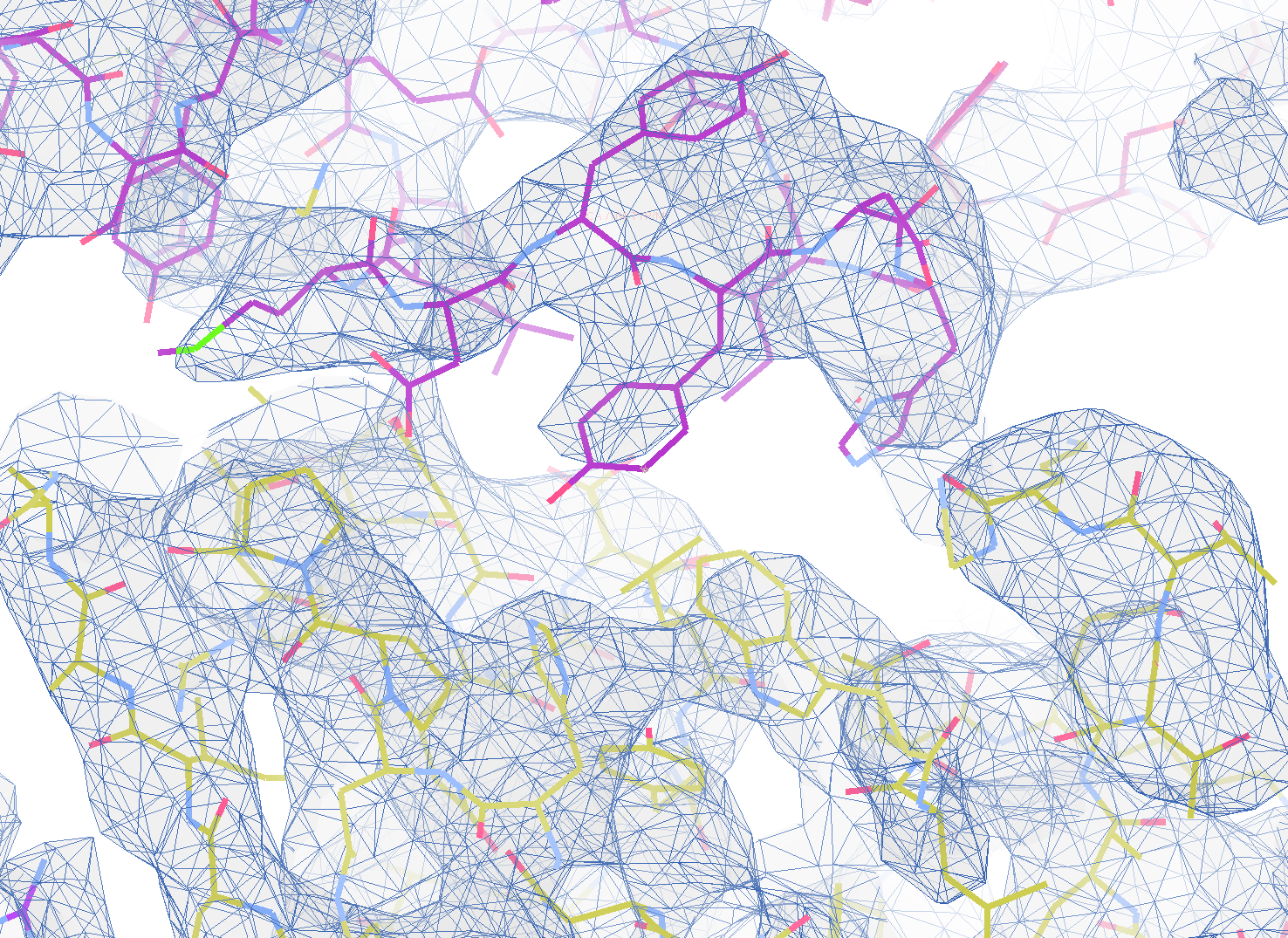

Supplement: Figure S3 — Representative electron density at the 1F1-HA interface, in the vicinity of CDR H3. The antibody and HA are depicted as magenta and yellow sticks, respectively. The 2FO-FC electron density map (blue mesh) is contoured at 1 sigma. (TIF) [file ppat.1003067.s003.tif]
