# Supplementary material for: Influenza Human Monoclonal Antibody 1F1 Interacts with Three Major Antigenic Sites and Residues Mediating Human Receptor Specificity in H1N1 Viruses
Source: PLoS Pathog. 2012 Dec 6;8(12):e1003067. doi: 10.1371/journal.ppat.1003067 (PMC3516549; doi:10.1371/journal.ppat.1003067)

Figure S2.

1F1 curve fittings

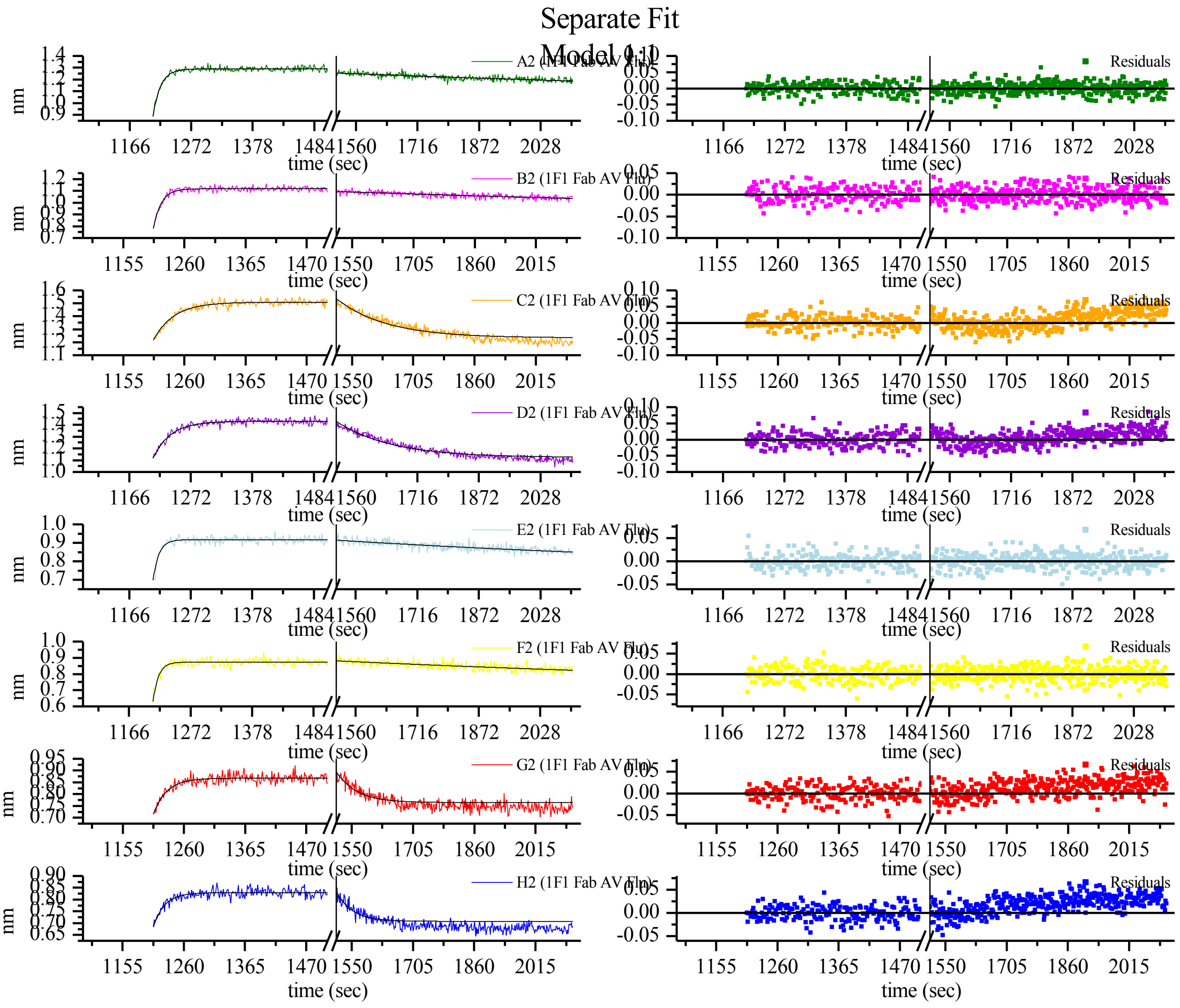

1I20 curve fittings

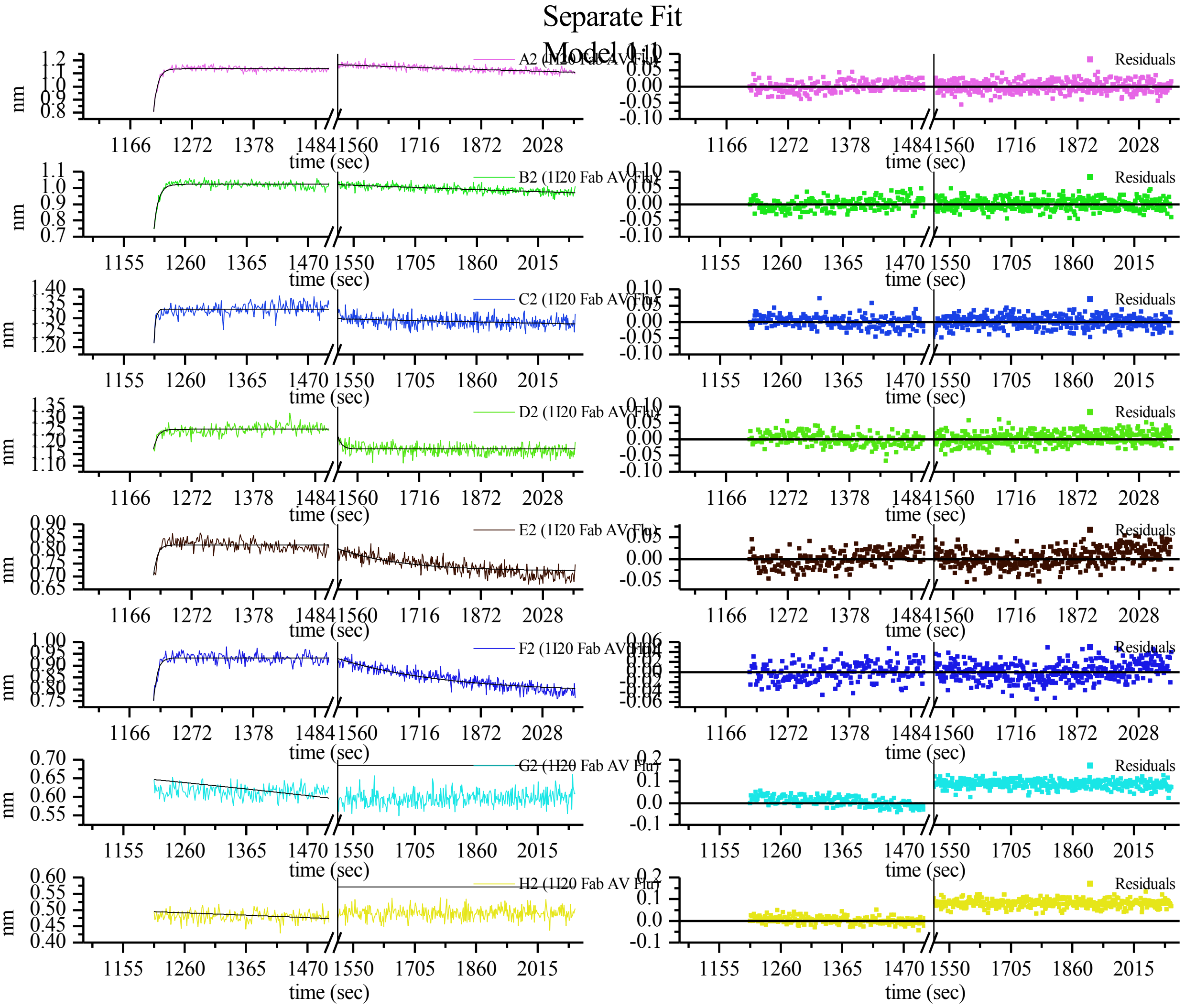

2B12 curve fittings

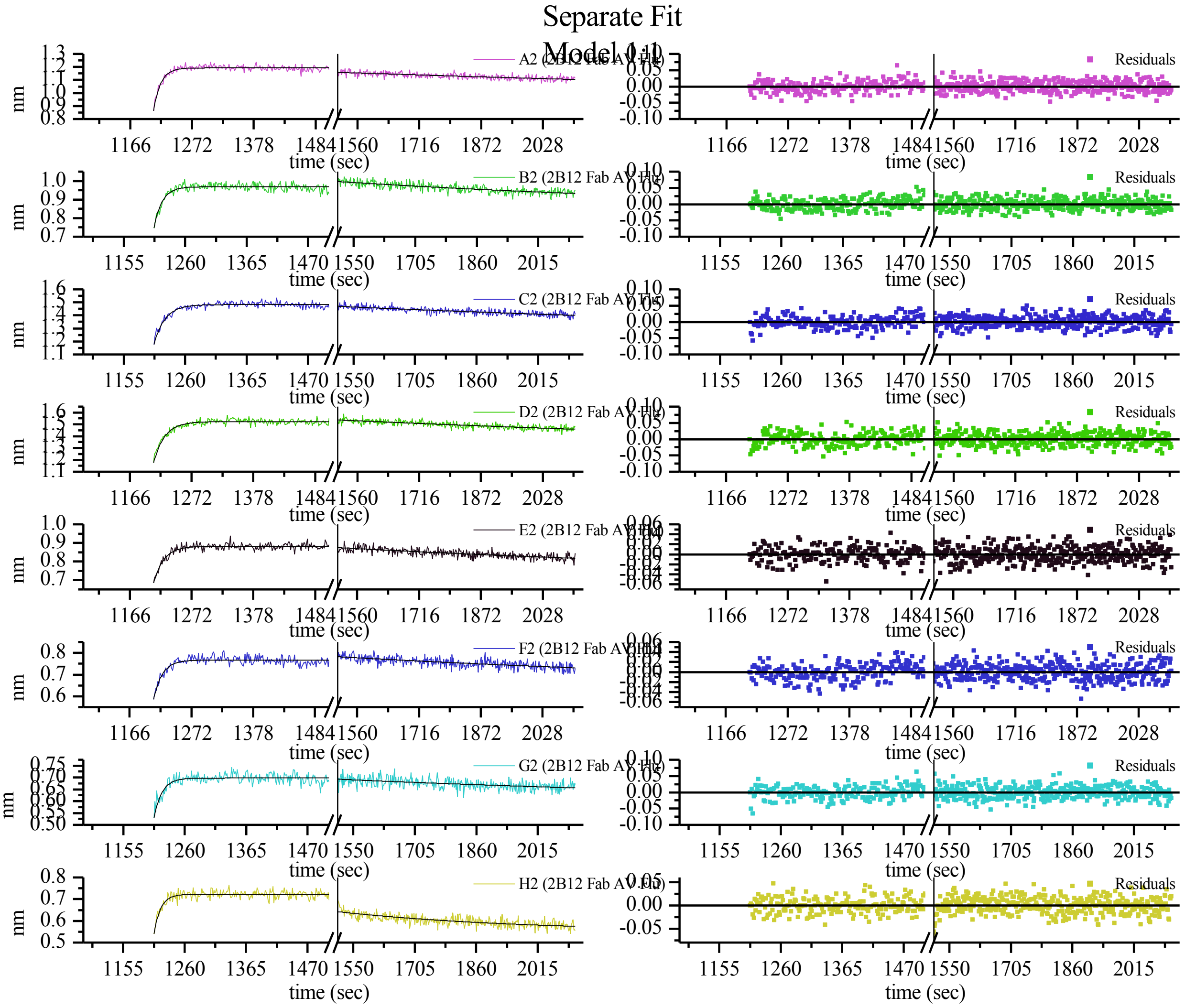

2D1 curve fittings

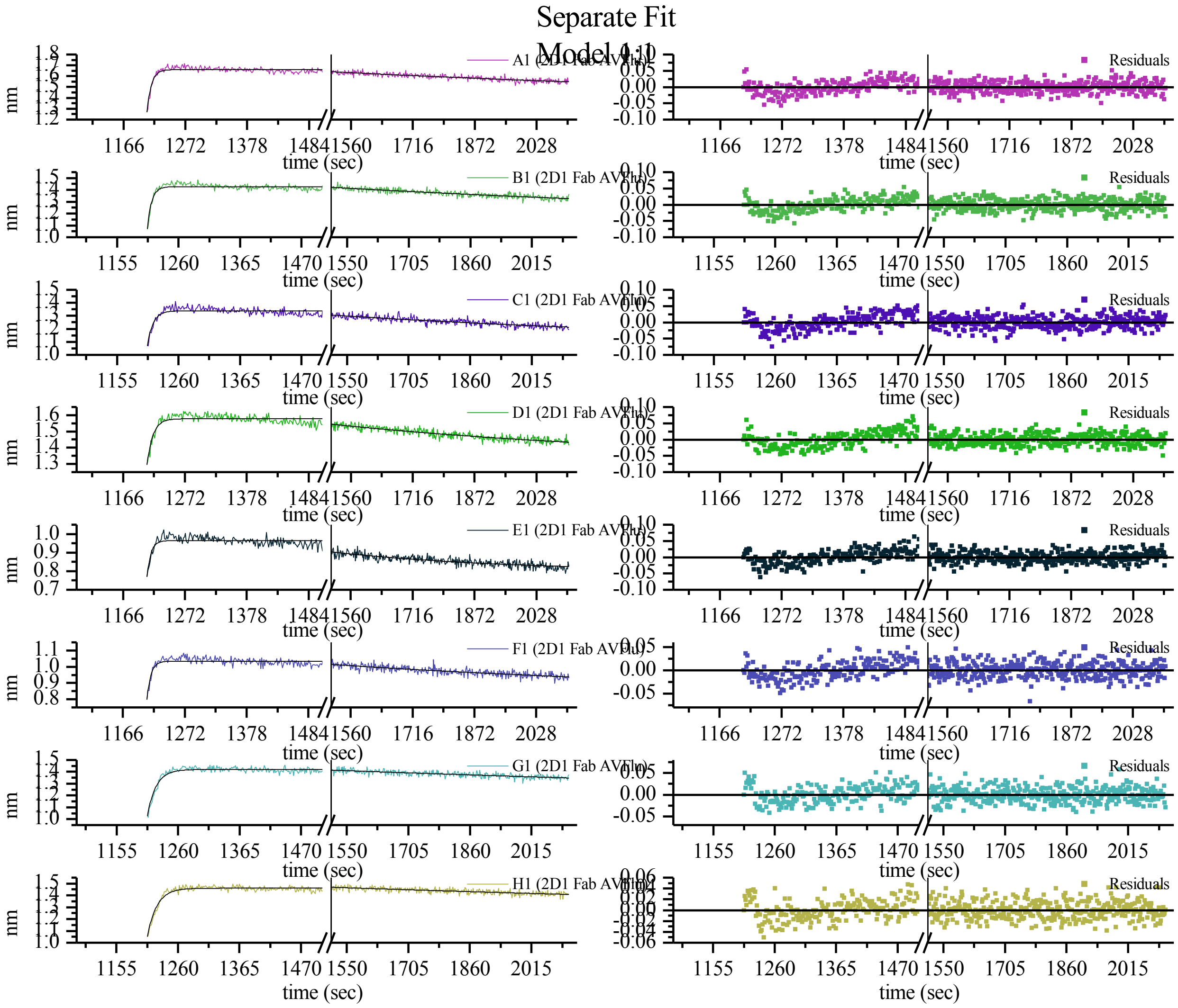

4D20 curve fittings

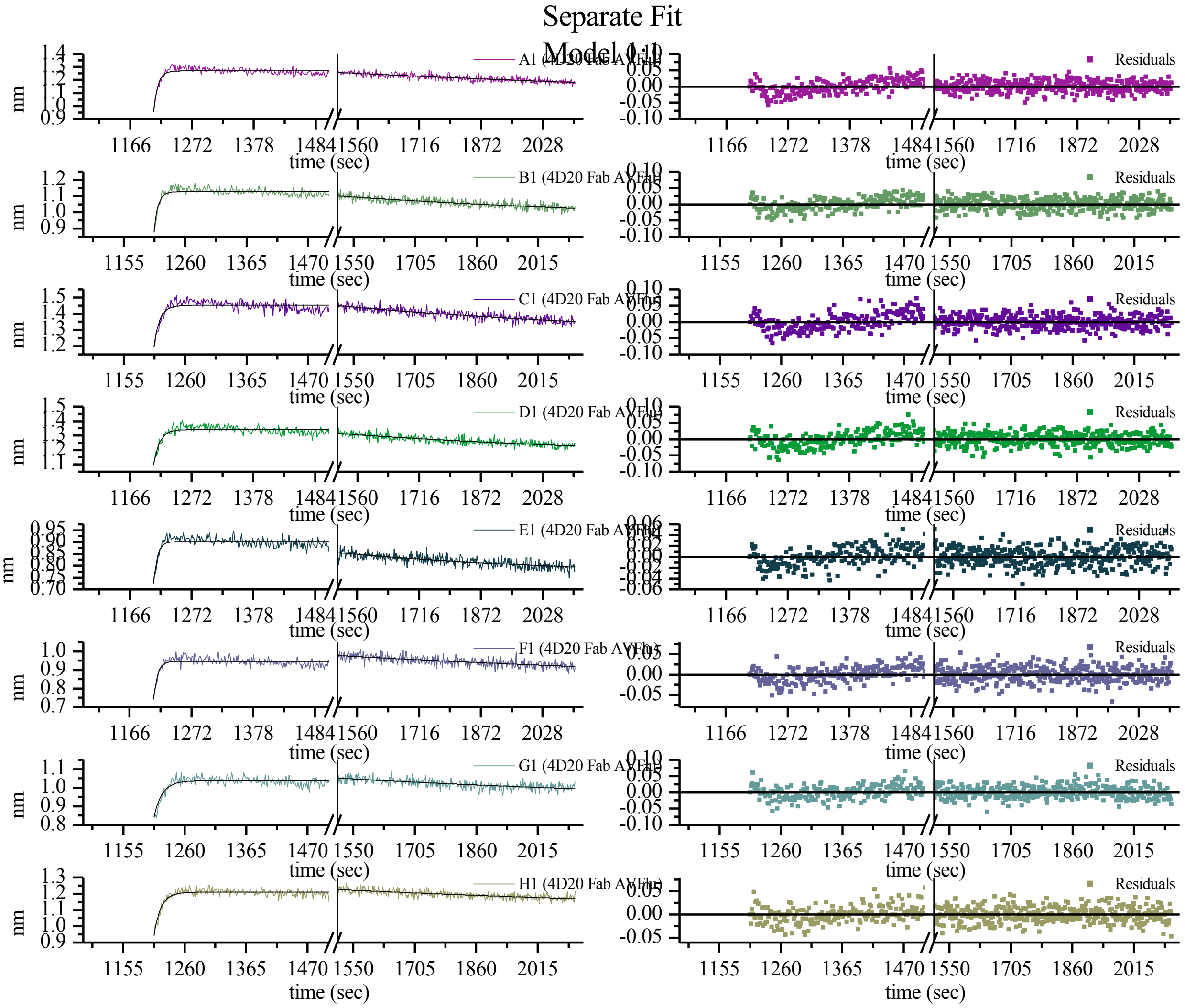

Supplement: Figure S2 — Automated curve fittings prompted by the Octet 4 software (ForteBio) of Fab 1F1, 1I20, 2B12, 2D1, or 4D20 affinities in association with the wild type SC1918 HA, a D190E variant, the D225G variant (NY1918), or the D190E/D225G double mutant (AV1918). (PDF) [file ppat.1003067.s002.pdf]
