# Supplementary material for: Influenza Human Monoclonal Antibody 1F1 Interacts with Three Major Antigenic Sites and Residues Mediating Human Receptor Specificity in H1N1 Viruses
Source: PLoS Pathog. 2012 Dec 6;8(12):e1003067. doi: 10.1371/journal.ppat.1003067 (PMC3516549; doi:10.1371/journal.ppat.1003067)
Supplement: Table S1 — Sequence alignment of 1F1 and 1I20 heavy chains (GenBank GI:309753504 and GI:163931316, respectively). The amino-acid residue numbering follows the Kabat scheme to be consistent with the numbering in other crystal structures in the PDB. The framework/loop definitions are based on the international ImMunoGeneTics information system (IMGT). (PDF) [file ppat.1003067.s004.pdf]

Table S1. Sequence alignment of 1F1 and 1I20 heavy chains (GenBank GI: 309753504 and GI:163931316, respectively). The amino-acid residue numbering follows the Kabat scheme to be consistent with the numbering in other crystal structures in the PDB. The framework/loop definitions are based on the international ImMunoGeneTics information system (IMGT).

FR=framework, CDR-H1-3=heavy chain complementarity determining regions 1, 2, or 3

```
                    FR1              CDR-H1      FR2         CDR-H2
         _________________________ ________ ________________ ________
                  10        20          30          40         50
         1234567890123456789012345 67890123 4567890123456789 12A34567
1F1H     EVQLVQSGGGVVQPRRSLRLSCAAS GFTFSSYA MHWVRQAPGKGLEWVAV ISYDGRNK
1I20H    .....E........GG......I.. A.NLGIFG ...............F .R...GKT

                        FR3                           CDR-H3           FR4
         ______________________________________ ____________________ __________
           60        70        80            90        100                 110
         89012345678901234567890123ABC3456789012 34567890ABCDEFGHI12  3456789012
1F1H     YYADSVKGRFTVSRDNSKNTLYLQMNSLRAEDTSVYYC ARELLMDYYDHIGYSPGPT- WGQGTLVTVS
1I20H    F..........I.................T...AL... ..DATA...GPGS.PNWLDP ..........
```
