# Supplementary material for: Influenza Human Monoclonal Antibody 1F1 Interacts with Three Major Antigenic Sites and Residues Mediating Human Receptor Specificity in H1N1 Viruses
Source: PLoS Pathog. 2012 Dec 6;8(12):e1003067. doi: 10.1371/journal.ppat.1003067 (PMC3516549; doi:10.1371/journal.ppat.1003067)
Supplement: Table S2 — Data collection and refinement statistics. (PDF) [file ppat.1003067.s005.pdf]

Table S2. Data collection and refinement statistics.

Data collection	1F1 Fab	Av1918 HA	1F1-SC1918 Complex
Beamline	SSRL 11-1	APS 23ID-D	SSRL 11-1
Wavelength (Å)	1.007	1.033	0.979
Space group	$P2_12_12_1$	$P2_1$	$P2_1$
Unit cell parameters (Å, °)	a=51.8, b=99.5, c=175.1, α=β=γ=90	a=71.8, b=241.5, c=72.0, α=γ=90, β=119.8	a=153.7, b=176.3, c=168.1, α=γ=90, β=92.1
Resolution (Å)	50-1.45 (1.55-1.45)[a]	50-1.80 (1.90-1.80)[a]	50-3.30[g] (3.42-3.30)[a]
Observations	1,316,517	719,448	368,013
Unique Reflections	160,778	193,879	108,722
Redundancy	6.5 (5.6)[a]	3.7 (2.8)[a]	3.4 (2.3)[a]
Completeness (%)	99.5 (97.9)[a]	98.5 (92.4)[a]	80.6 (34.9)[a]
$<I/\sigma_I>$	10.4 (2.4)[a]	15.9 (2.2)[a]	14.2 (1.9)[a]
R_{sym}[b]	0.07 (0.58)[a]	0.09 (0.57)[a]	0.06 (0.52)[a]
Z_a[c]	2	3	6
Refinement statistics			
Resolution (Å)	50-1.45	50-1.80	50-3.30
Reflections (work)	149,459	179,611	102,884
Refections (test)	7,896	9,533	5,428
R_{cryst}(%)[d] / R_{free}(%)[e]	18.1 / 20.7	18.0 / 20.9	22.2 / 26.0
Average B (Å^2)	34.3	51.5	95.2
Wilson B (Å^2)	22.3	30.3	98.8
Protein atoms	6,792	11,825	38,010
Carbohydrate atoms	0	220	402
Waters	740	897	0
RMSD from ideal geometry			
Bond length (Å)	0.011	0.011	0.04
Bond angles (°)	1.32	1.27	0.92
Ramachandran statistics (%)[f]			
Favored	97.6	97.7	96.3
Outliers	0.1	0.0	0.0
PDB Code[g]	4GXV	4GXX	4GXU

[a] Numbers in parentheses refer to the highest resolution shell.
[b] $R_{sym} = \Sigma_{hkl} \Sigma_i | I_{hkl,i} - <I_{hkl}> | / \Sigma_{hkl} \Sigma_i I_{hkl,I}$, where $I_{hkl,i}$ is the scaled intensity of the ith measurement of reflection h, k, l, $< I_{hkl} >$ is the average intensity for that reflection, and n is the redundancy [1].
[c] Z_a is the number of HA monomers, Fabs or HA monomer-Fab complexes per crystallographic asymmetric unit.
[d] $R_{cryst} = \Sigma_{hkl} | F_o - F_c | / \Sigma_{hkl} | F_o |$ x 100
[e] R_{free} was calculated as for R_{cryst}, but on a test set comprising 5% of the data excluded from refinement.
[f] Calculated using Molprobity [2].
[g] Since the 1F1-SC1918 dataset is incomplete to 3.3 Å due to anisotropic diffraction (~81% complete overall and ~35% complete in the high resolution shell), we report the effective resolution to be 3.55 Å, as suggested by Hazes [3]. This corresponds to the resolution of a dataset that is 100% complete and has the same number of reflections as observed in the current dataset. There are 11,043 observed reflections between 3.55 and 3.30 Å (40.7% completeness for this bin) included

in the refinement.

References

1. Weiss MS, Hilgenfeld R (1997) On the use of the merging R factor as a quality indicator for X-ray data. J Appl Crystallog 30: 203-205.
2. Chen VB, Arendall WB, 3rd, Headd JJ, Keedy DA, Immormino RM, et al. (2010) MolProbity: all-atom structure validation for macromolecular crystallography. Acta crystallographica Section D, Biological crystallography 66: 12-21.
3. Kleywegt GJ (2000) Validation of protein crystal structures. Acta crystallographica Section D, Biological crystallography 56: 249-265.
