# Supplementary material for: Influenza Human Monoclonal Antibody 1F1 Interacts with Three Major Antigenic Sites and Residues Mediating Human Receptor Specificity in H1N1 Viruses
Source: PLoS Pathog. 2012 Dec 6;8(12):e1003067. doi: 10.1371/journal.ppat.1003067 (PMC3516549; doi:10.1371/journal.ppat.1003067)
Supplement: Table S3 — Affinity measurement data of Fabs 1F1, 1I20, 2B12, 2D1, or 4D20 in association with the wild type SC1918 HA, a D190E variant, the D225G variant (NY1918), or the D190E/D225G double mutant (AV1918). (PDF) [file ppat.1003067.s006.pdf]

Table S3. Affinity measurement data of Fabs 1F1, 1I20, 2B12, 2D1, or 4D20 in association with the wild type SC1918 HA, a D190E variant, the D225G variant (NY1918), or the D190E/D225G double mutant (AV1918). Data were calculated using Origin 7.5 SR6 software (OriginLab Corp., Northampton, MA) based on automated curve fittings prompted by the Octet 4 software (ForteBio). All measurements were performed in duplicate.

Fab	Antigen	Molar Conc [M]	k_{obs} [1/s]	Error in k_{obs}	k_d [1/s]	Error in k_d	k_a [1/Ms]	K_D [M]	Assoc R^2	Assoc Chi^2	Dissoc R^2	Dissoc Chi^2
1F1	SC1918	1.2E-6	7.62E-2	2.36E-3	3.72E-4	1.65E-5	6.32E4	5.89E-9	0.92707	2.84466E-4	0.57396	2.85517E-4
		1.2E-6	7.36E-2	2.72E-3	4.02E-4	1.93E-5	6.10E4	6.59E-9	0.90423	2.97915E-4	0.53747	2.55073E-4
	D190E	1.2E-6	2.85E-2	9.64E-4	7.95E-3	2.34E-4	1.71E4	4.65E-7	0.91977	3.71098E-4	0.8981	7.9874E-4
		1.2E-6	3.36E-2	1.19E-3	7.22E-3	1.65E-4	2.20E4	3.28E-7	0.90656	4.23497E-4	0.92889	5.3838E-4
	D225G (NY1918)	1.2E-6	9.93E-2	5.85E-3	7.73E-4	3.20E-5	8.21E4	9.41E-9	0.8103	2.38142E-4	0.61271	2.19514E-4
		1.2E-6	1.06E-1	6.88E-3	5.30E-4	2.76E-5	8.81E4	6.01E-9	0.73835	3.37669E-4	0.49776	2.88267E-4
	DM (AV1918)	1.2E-6	4.34E-2	2.82E-3	1.93E-2	1.63E-3	2.00E4	9.64E-7	0.74024	2.88112E-4	0.60227	5.32857E-4
		1.2E-6	5.13E-2	3.39E-3	2.49E-2	2.84E-3	2.20E4	1.13E-6	0.7205	2.38373E-4	0.40671	7.23607E-4
1I20	SC1918	1.2E-6	1.40E-1	6.54E-3	3.75E-4	1.86E-5	1.16E5	3.22E-9	0.86042	2.51372E-4	0.52242	2.60006E-4
		1.2E-6	1.27E-1	7.72E-3	3.66E-4	2.13E-5	1.06E5	3.46E-9	0.79398	3.18227E-4	0.44185	2.68696E-4
	D190E	1.2E-6	3.40E-1	7.76E-2	3.86E-4	6.77E-5	2.83E5	1.36E-9	0.23448	3.28174E-4	0.07826	2.96718E-4
		1.2E-6	1.40E-1	2.99E-2	1.04E-1	3.18E-2	3.03E4	3.43E-6	0.23936	3.30283E-4	0.08623	3.5408E-4
	D225G (NY1918)	1.2E-6	1.62E-1	2.79E-2	6.24E-3	4.23E-4	1.30E5	4.80E-8	0.37887	3.66524E-4	0.61016	4.17659E-4
		1.2E-6	1.55E-1	1.58E-2	3.72E-3	1.17E-4	1.26E5	2.94E-8	0.56951	3.23466E-4	0.81217	3.2196E-4
	DM (AV1918)	1.2E-6	-4.25E-4*	1.94E-3	0*	0	--	--	-0.91544	4.93356E-4	-24.27658	0.00811
		1.2E-6	-4.31E-4*	3.38E-3	0*	0	--	--	-0.18168	2.71785E-4	-22.97051	0.00688
2B12	SC1918	1.2E-6	8.19E-2	3.31E-3	3.51E-4	1.85E-5	6.79E4	5.17E-9	0.87809	3.03955E-4	0.49004	2.66011E-4
		1.2E-6	7.22E-2	4.22E-3	8.59E-4	3.83E-5	5.95E4	1.44E-8	0.79029	3.22055E-4	0.57888	2.50456E-4
	D190E	1.2E-6	5.31E-2	2.00E-3	5.72E-4	2.48E-5	4.38E4	1.31E-8	0.88294	3.29169E-4	0.59077	2.83465E-4
		1.2E-6	5.32E-2	1.96E-3	4.59E-4	1.93E-5	4.40E4	1.04E-8	0.88458	4.04956E-4	0.60465	3.39483E-4
	D225G (NY1918)	1.2E-6	5.83E-2	2.90E-3	6.87E-4	3.24E-5	4.80E4	1.43E-8	0.82814	2.25363E-4	0.54984	2.28666E-4
		1.2E-6	6.82E-2	4.26E-3	6.88E-4	3.86E-5	5.62E4	1.22E-8	0.75661	2.56576E-4	0.46407	2.56015E-4
	DM (AV1918)	1.2E-6	7.38E-2	6.10E-3	6.79E-4	6.12E-5	6.10E4	1.11E-8	0.60121	3.6777E-4	0.24982	3.40923E-4
		1.2E-6	8.85E-2	6.40E-3	2.18E-3	1.10E-4	7.19E4	3.03E-8	0.69873	2.81884E-4	0.53768	3.11043E-4
2D1	SC1918	6E-7	1.32E-1	6.58E-3	5.34E-4	1.64E-5	2.18E5	2.44E-9	0.8551	4.27772E-4	0.74237	2.54932E-4
		6E-7	1.36E-1	7.73E-3	5.84E-4	1.83E-5	2.26E5	2.58E-9	0.8253	4.47122E-4	0.73554	2.77782E-4
	D190E	6E-7	1.03E-1	7.95E-3	8.40E-4	2.90E-5	1.70E5	4.95E-9	0.71168	6.09305E-4	0.69968	3.19243E-4
		6E-7	1.00E-1	7.10E-3	1.10E-3	2.97E-5	1.65E5	6.68E-9	0.73563	5.84568E-4	0.79253	2.70292E-4
	D225G (NY1918)	6E-7	1.51E-1	1.68E-2	2.25E-3	8.80E-5	2.48E5	9.09E-9	0.55358	4.60279E-4	0.68221	2.4953E-4
		6E-7	1.51E-1	1.28E-2	8.30E-4	3.23E-5	2.50E5	3.31E-9	0.67316	3.87994E-4	0.64372	2.72639E-4
	DM (AV1918)	6E-7	6.72E-2	2.40E-3	3.49E-4	1.59E-5	1.11E5	3.13E-9	0.91684	4.0514E-4	0.56453	2.85787E-4
		6E-7	6.15E-2	1.93E-3	3.15E-4	1.47E-5	1.02E5	3.09E-9	0.93215	3.6698E-4	0.5514	2.59621E-4

**4D20**	**SC1918**	6E-7	1.37E-1	8.97E-3	5.30E-4	2.01E-5	2.27E5	2.34E-9	0.766	4.70195E-4	0.65213	2.66628E-4
		6E-7	1.41E-1	1.11E-2	9.09E-4	3.77E-5	2.34E5	3.88E-9	0.71319	4.07952E-4	0.61533	3.05514E-4
	**D190E**	6E-7	1.01E-1	9.21E-3	1.10E-3	3.98E-5	1.67E5	6.60E-9	0.63313	7.54765E-4	0.68181	3.75513E-4
		6E-7	9.63E-2	7.78E-3	1.13E-3	4.42E-5	1.59E5	7.14E-9	0.67313	5.81006E-4	0.64641	3.47705E-4
	**D225G (NY1918)**	6E-7	1.54E-1	1.56E-2	1.44E-3	6.99E-5	2.54E5	5.64E-9	0.59987	3.0875E-4	0.55383	2.56643E-4
		6E-7	1.48E-1	1.41E-2	7.48E-4	4.17E-5	2.46E5	3.04E-9	0.61551	3.79996E-4	0.46799	3.35474E-4
	**DM (AV1918)**	6E-7	7.09E-2	5.42E-3	8.85E-4	4.89E-5	1.17E5	7.58E-9	0.72492	4.31301E-4	0.47115	3.02938E-4
		6E-7	8.46E-2	4.53E-3	6.77E-4	3.53E-5	1.40E5	4.84E-9	0.82016	3.40109E-4	0.49878	2.70188E-4
