# Supplementary material for: Influenza Human Monoclonal Antibody 1F1 Interacts with Three Major Antigenic Sites and Residues Mediating Human Receptor Specificity in H1N1 Viruses
Source: PLoS Pathog. 2012 Dec 6;8(12):e1003067. doi: 10.1371/journal.ppat.1003067 (PMC3516549; doi:10.1371/journal.ppat.1003067)
Supplement: Table S4 — 1F1-HA contacts. Summary of interacting residue pairs from chains A, B, M, and N in the 1F1-Sc1918 crystal structure, generated using CONTACSYM [42]. (PDF) [file ppat.1003067.s007.pdf]

Table S4. 1F1-HA contacts. Summary of interacting residue pairs from chains A, B, M, and N in the 1F1-Sc1918 crystal structure, generated using CONTACSYM [1].

1F1 Residue	HA Residue
VH-SER31	LYS222
VH-TYR32	THR187
VH-TYR52A	LYS222
	ASP225
VH-LEU96	THR189
VH-LEU97	THR189
	ASP190
	SER193
VH-MET98	PRO186
	THR187
	ASP190
	LYS222
	ALA227
VH-ASP99	PRO186
	ASP190
	LYS222
	ALA227
	GLY228
VH-TYR100A	TRP153
	HIS183
	LEU194
VH-ASP100B	VAL135
	SER145
VH-HIS100C	LEU194
	LYS133A
VH-ILE100D	SER193
	LEU194
VL-TYR49	THR189
	GLN192
	SER193
VL-SER50	LYS156
	SER193
VL-LEU51	SER159

VL-ASN52	SER159
	GLN196
VL-GLN53	GLN192
	GLN196

Reference

1. Sheriff S, Hendrickson WA, Smith JL (1987) Structure of myohemerythrin in the azidomet state at 1.7/1.3 A resolution. Journal of molecular biology 197: 273-296.
